# Physical frailty and long-term mortality in older people with chronic heart failure with preserved and reduced ejection fraction: a retrospective longitudinal study

**DOI:** 10.1186/s12877-020-01971-4

**Published:** 2021-02-01

**Authors:** Shuo-Chun Weng, Chu-Sheng Lin, Der-Cherng Tarng, Shih-Yi Lin

**Affiliations:** 1 Institute of Clinical Medicine, School of Medicine, National Yang Ming Chiao Tung University, Taipei, Taiwan; 2 Center for Geriatrics and Gerontology, Taichung Veterans General Hospital, Taichung, Taiwan; 3 Division of Nephrology, Department of Internal Medicine, Taichung Veterans General Hospital, Taichung, Taiwan; 4 Department of Family Medicine, Taichung Veterans General Hospital, Taichung, Taiwan; 5 Department and Institute of Physiology, National Yang Ming Chiao Tung University, Taipei, Taiwan; 6 Division of Nephrology, Department of Medicine, Taipei Veterans General Hospital, Taipei, Taiwan; 7 Center for intelligent Drug Systems and Smart Bio-devices (IDS2B), Hsinchu, Taiwan; 8 Department of Biological Science and Technology, College of Biological Science and Technology, National Yang Ming Chiao Tung University, Hsinchu, Taiwan; 9 Center for Geriatrics and Gerontology, Division of Endocrinology and Metabolism, Department of Internal Medicine, Taichung Veterans General Hospital, No.1650 Boulevard Sect. 4, Taichung, Taiwan

**Keywords:** All-cause mortality, Charlson comorbidity index, Function reserve, Handgrip strength, Heart failure, Timed up and go test

## Abstract

**Background:**

Frailty, a syndrome characterized by a decline in function reserve, is common in older patients with heart failure (HF) and is associated with prognosis. This study aimed to evaluate the impact of frailty on outcomes in older patients with preserved and reduced cardiac function.

**Methods:**

In total, 811 adults aged ≥65 years were consecutively enrolled from 2009 to 2018. HF was diagnosed according to the ICD9 code and a 2D echocardiogram was categorized by reduced ejection fraction (HFrEF) and preserved ejection fraction (HFpEF). The index date was registered at the time of HF. All patients received a comprehensive geriatric assessment, and clinical outcomes were examined with adjustment of the other prognostic variables.

**Results:**

Mean age was 80.5 ± 7.1 years. The prevalence of HF, HFpEF, HFrEF, Fried, and Rockwood frailty indicators was 28.5, 10.4, 9.7, 52.5, and 74.9%, respectively. At baseline, scores in the Timed Up and Go test was closely associated with the severity of HF, either with HFpEF or HFrEF. After a mean follow-up of 3.2 ± 2.0 years, we found that HF patients with low handgrip strength (HGS) had the poorest survival, followed by non-HF patients with decreased HGS, and HF with fair HGS in comparison with non-HF with fair HGS (*p* = 0.008) if participants were arbitrarily divided into two HGS groups. In all patients, a high Rockwood frailty index was independently associated with increased mortality (adjusted hazard ratio [aHR] = 1.05; 95% confidence interval [CI]: 1.0004 to 1.10). In addition, the adjusted mortality HR was 3.42 with decreased HGS (95% CI: 1.03 to 11.40), 7.65 with use of mineralocorticoid receptor antagonist (95% CI: 2.22 to 26.32), and 1.26 with associated multi-comorbidities assessed by Charlson comorbidity index (95% CI: 1.05 to 1.51).

**Conclusions:**

Our study results indicate that frailty and decreased physical functions were associated with HF. Besides, frailty and HGS predicted prognosis in the patients, and there was a combined effect of HF and low HGS on survival.

**Supplementary Information:**

The online version contains supplementary material available at 10.1186/s12877-020-01971-4.

## Background

Heart failure (HF) is a classical representation of the aging process with diminished physiologic reserve of cardiac function and increased vulnerability to external or internal challenges [1]. As populations age worldwide, there is a constant increase in the incidence of HF, which has become one of the biggest challenges in modern cardiology. According to data from Taiwan’s Society of Cardiology, the HF with reduced Ejection Fraction (TSOC-HFrEF) registry, and Taiwan’s National Health Insurance Research Database, older adults have a 25-fold higher risk of HF hospitalization, the longer length of stay, higher total medical expense, higher in-hospital mortality, and comprise 63.9% of all deaths from cardiovascular (CV) origin during 1-yr follow-up in comparison with younger people [2].

In aging populations with HF, there is an increase in concomitant non-cardiac conditions, which can complicate management and contribute to adverse outcomes [3]. Among these comorbidities, frailty, a syndrome caused by multisystem dysregulations, impaired homeostasis, and decreased physiologic reserve, frequently occurs in HF patients with a prevalence ranging from 15 to 74%, depending on the studied population and the method of assessment [4–6]. In older adults hospitalized for HF, it has been shown that poor physical and mental functioning, as well as frailty, increase the risk of hospital readmission, institutionalization, and death [7]. Moreover, several frailty indicators, including gait speed (GS > 1.0 m/sec) [8], Timed Up and Go (TUG) test scores [7], and handgrip strength (HGS) [8] were each reported to be associated with prognosis in patients with HF.

However, despite the large number of studies showing an association between frailty and HF in older adults, few studies to date have evaluated other factors concurrently, such as cognition disorder, malnutrition, and different physical functional status, which also affect the prognosis of HF patients [9]. Thus, the present study analyzed the results of the comprehensive geriatric assessment (CGA), which evaluates socioeconomic, comorbidity, polypharmacy, physical and cognitive functions, mood, and nutrition, to determine the effects of frailty and/or other geriatric disorders on outcomes in older adults with HF. Furthermore, a recent meta-analysis found median prevalence rates of left ventricular diastolic dysfunction and diastolic HF in older adults (> 60 years) of 36.0 and 4.9%, respectively, and these patients now constitute the dominant subset of HF in older people [10]. Therefore, this study also aimed to determine the prevalence of frailty and outcomes in older patients with HFrEF and HFpEF.

## Methods

### Study design and participants

**Fig. 1:**
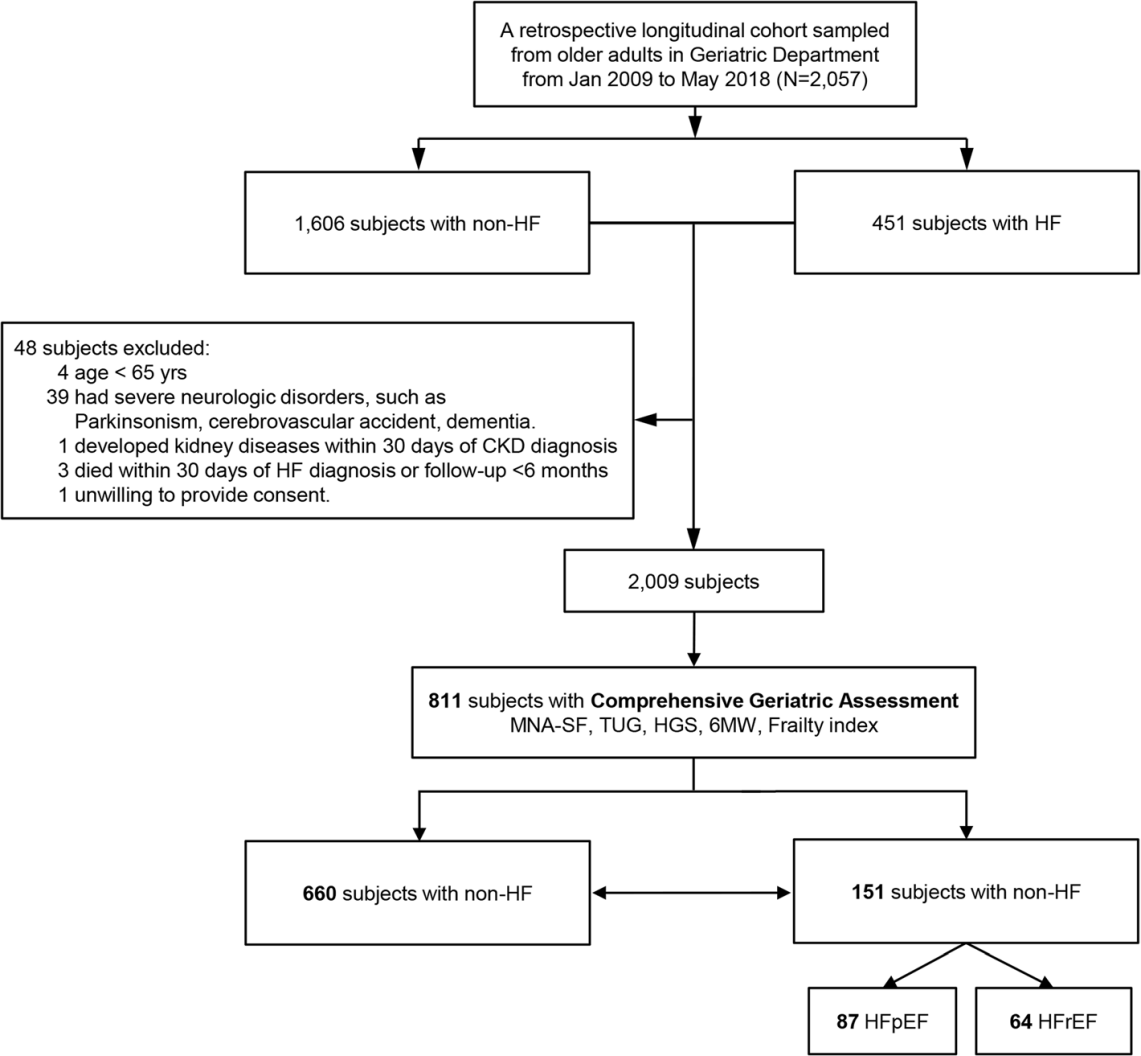
Flowchart presenting the selected participants. 151 patients had heart failure (HF), and 660 subjects had non-HF. *Abbreviations: MNA-SF* mini-nutritional assessment-short form; *TUG* timed up and go test; *HGS* handgrip strength; *6 MW* 6-m walking

For this retrospective tertiary hospital-based cohort, we retrieved all data from a registered disease management system, and all individuals (*N* = 2057) were aged ≥65 yrs. and selected for the period Jan 2009 to May 2018 from Taichung Veterans General Hospital (TCVGH; Fig. 1). The participants were (a) individuals who visited the inpatient and outpatient (OPD) of the geriatric department, (b) older adults attending community-based healthcare screening programs, and (c) residents in a veterans’ home. The exclusion criteria were age < 65 yrs., complicated with severe neurologic disorders, and death within 30 days of HF diagnosis or follow-up < 6 months. Finally, 811 study participants (660 non-HF and 151 HF patients, with 87 HFpEF and 64 HFrEF) were enrolled and concurrently underwent standard laboratory tests and echocardiography. All patients’ general demographic data were recorded in medical history by the physicians. Data included age, gender, body mass index, lifestyle habits, education, marital status, and socioeconomic status. We also assessed their medical histories and comorbid conditions and recorded their diagnosed diseases, medications, and their Charlson Comorbidity Index (CCI) [7].

Moreover, all enrolled patients received standardized measures of CGA, including mini-mental state examination (MMSE), 5-item geriatric depression scale (GDS-5), activities of daily living (ADL), instrumental activities of daily living (IADL), mini-nutritional assessment (MNA), handgrip strength (HGS), walking speed (WS) and timed up and go test (TUG), by trained nurses. The frailty was classified as robust, prefrail, or frail, based on five components: weakness, slowness, exhaustion, low activity, and weight loss according to Fried criteria [11], in which reference values of HGS and 6 MW were assessed according to previously published Asia-Pacific clinical practice guidelines [12]. Informed consent was not required from the enrolled subjects, and the study protocol conforms to the ethical guidelines of the 1975 Declaration of Helsinki as reflected in a priori approval by the Institutional Review Board (IRB) of TCVGH (No.CF13015, CF13015–1, CF13015–2, CF13015–3).

### Diagnosis of heart failure

The diagnosis and stages of HF, including congestive or systolic HF, diastolic heart failure, or cardiomyopathy adhered to the criteria of the American College of Cardiology Foundation / American Heart Association (ACC/AHA) [13]. Chronic heart failure (CHF) was defined by the International Classification of Diseases, Ninth Revision, Clinical Modification (ICD-9-CM) codes (428.0–428.9, 402.91), and 2D echocardiogram according to the LV ejection fraction (LVEF) ≥50% and LVEF < 50% were denoted by the abbreviations HFpEF and HFrEF, respectively. Functional classification of HF was stratified as class 0, A, B, C, and D by the ACC/AHA guideline [13]. The other relevant cardiovascular diseases, atrial fibrillation (AF) with code 427.31 and cardiac arrhythmia with codes 427.0–427.9, were also recorded. Concomitant medication use with HF was recorded according to the ATC code (supplemental data – Appendix 1).

### Frailty index

Both Fried criteria [11] and Rockwood frailty index [14] were used to detect frailty in older people to measure frailty with the pathobiological link with HF and mortality. A modified Rockwood frailty index was created by utilizing health deficits that are collected in health assessments, including 21 chronic diseases, 4 items (MNA-SF, TUG, HGS, 6 MW) of CGA, and 15 abnormal laboratory data. Categories were created according to established cutoffs in community-dwelling cohorts to match the Fried physical phenotype: non-frail (0–0.1), prefrail (> 0.1–0.21), and frail (> 0.21) [15]. The Kappa statistic was used to assess agreement between the Fried and Rockwood frailty phenotype, for which 95% confidence intervals (95% CI) were estimated.

### Procedures of benchmarking research in physical functionality correlated with patients with heart failure

Because of the poor correlations of MNA-SF, TUG, HGS, 6 MW, and frailty index (area under the receiver operating characteristic [AUC]: 0.517, 0.569, 0.404, 0.605, and 0.508, respectively) with HF and disease severity when using the traditional cut-off point for TUG, HGS, and 6 MW according to previous studies [12, 16], the present study tried to define new cut-off points in the various frailty parameters. We arbitrarily separated TUG values into fifths and used the Chi-square test to determine the appropriate cut-off point of 25 s (secs). The HGS was originally different in both genders. Hence HGS values were divided into tertiles, and the cut-off point was 20.4 kg (kgs) in men and 15.4 kgs in women, respectively. Gait speed was also different between genders and varied based on leg length. Thus, 6 MW values were calculated into deciles, with a cut-off point of 25 s in men and eight equal parts with a cut-off point of 23 s in women.

### Study outcome and follow-up

The index date was defined as the date of HF diagnosis. CGA and 2D echocardiogram were completed nearby the diagnosis of HF. The studied outcome was all-cause mortality obtained from the Clinical Information Research & Development Center, TCVGH, and the accuracy of death was validated by Taiwan’s National Death Registry according to the ICD-9 (ICD9 001.x-999.x) or ICD10 (A00.x-Z99.x). All participants were followed until death or June 19, 2018 to prevent lead-time bias.

### Statistical analysis

Continuous variables were analyzed by the Mann-Whitney U test with mean ± standard deviation (SD) or median with interquartile range (IQR). Categorical data by Chi-square tests/Fisher’s exact test were expressed as numbers and percentages. MNA-SF, TUG, HGS, and 6 MW of the study participants were stratified by tertile, equal eight, decile, etc., and analyzed by χ^2^ test. Kaplan-Meier (KM) plots were generated to estimate the cumulative survival rate. Then, Cox proportional hazards models were applied to multivariate analyses to estimate the hazard ratios of study outcome after adjusting for age, BMI, HFrEF, CCI, geriatric assessment, laboratory data, and medication. A *p* value for nonlinearity was calculated using a null hypothesis test. Statistical significance was set at *p* < 0.05. Statistical analyses were performed with SPSS for Windows version 22.0 (SPSS Institute Inc., Chicago, USA).

## Results

### Prevalence of clinical characteristics of the study population

Before the inclusion of 811 participants with complete CGA in the final analysis, a total of 2009 individuals were evaluated for the prevalence of HF. The prevalence of HF in the geriatric ward (25.3%) was high from 2009 to 2018 compared with the rate in the geriatric OPD (14.7%); however, the prevalence of HFrEF was low in both the geriatric OPD (6.7%) and the geriatric ward (10.7%) compared with HFpEF (8.0% in OPD; 14.6% in the ward) (supplemental data – Appendix 2). The baseline characteristics of the 811 participants with and without HF are listed in Table 1. Among the HF group, functional classification of HF was stage 0 0.0%, stage A 41.7%, stage B 36.4%, stage C 17.9%, and stage D 4.0%, respectively. In both groups, they had similar distributions of age, gender, diabetes mellitus, hyperlipidemia, low-density lipoprotein (LDL), hemoglobin, hemoglobin A1c (HbA1c), MNA scores, HGS, 6 MW, and frailty index. Compared with non-HF patients, HF patients had higher body mass index (BMI), a higher percentage of hypertension (HTN), cardiovascular disease, AF, myocardial infarction, chronic obstructive pulmonary disease (COPD), and CCI score, as well as poor LVEF, higher cardiac arrhythmia, significantly longer TUG test, high level of N-terminal pro-B-type natriuretic peptide (NT-proBNP), and high serum creatinine, but lower serum albumin and eGFR. The percentage of patients receiving medication for HF was high in the HF group (Table 1). Among different frailty assessment tools, the Fried criteria revealed a proportion of 52.5% of patients as frail, and the Rockwood frailty assessment tool found a proportion of 79.4% as frail (Table 1). There was low agreement between the two frailty tools (Kappa value of 0.0091).

**Table 1:** Baseline characteristics of older patients without and with heart failure

Characteristics	Non-Heart Failure (***n*** = 660)		Heart Failure (***n*** = 151)		***P*** value
Mean age (years)	81.4	(74.7–85.5)	82.7	(77.1–86.1)	0.116
Male	439	(66.5)	111	(73.5)	0.118
BMI (kg/m^2^)	23.8	(21.5–26.7)	24.5	(22.3–27.3)	0.030
Comorbidities					
Diabetes mellitus	341	(51.7)	84	(55.6)	0.430
Hypertension	554	(83.9)	138	(91.4)	0.027
Hyperlipidemia	277	(42.0)	74	(49.0)	0.138
Cardiovascular disease	150	(22.7)	65	(43.0)	< 0.001
AF	14	(2.1)	14	(9.3)	< 0.001
Myocardial infarction	0	(0.0)	86	(57.0)	< 0.001
COPD	252	(38.2)	79	(52.3)	0.002
CCI	1.0	(1.0–2.0)	2.0	(1.0–2.3)	0.002
LVEF	60.0	(56.0–62.0)	52.0	(42.0–59.0)	< 0.001
Cardiac arrhythmia	37	(5.6)	21	(13.9)	0.001
Geriatric assessment					
MNA-SF (0 to 14)	13.0	(11.0–14.0)	13.0	(11.0–14.0)	0.572
Timed Up and Go test (sec, IQR)	17.0	(12.3–22.0)	18.0	(14.0–28.0)	0.003
Handgrip strength (kg, IQR)	20.2	(15.0–25.2)	19.4	(15.1–26.9)	0.970
6-min walking test (sec, IQR)	11.0	(7.4–16.0)	11.0	(7.6–19.9)	0.792
Fried frailty index^a^					0.059
Robust	66	(10.0)	29	(19.3)	
Pre-frail	243	(36.8)	47	(31.3)	
Frail	351	(53.2)	75	(49.4)	
Rockwood frailty index^b^	27.0	(21.6–32.4)	36.8	(31.6–42.9)	< 0.001
Non-frail	11	(1.7)	0	(0.0)	< 0.001
Pre-frail	153	(23.2)	3	(2.0)	
Frail	496	(75.2)	148	(98.0)	
Laboratory data					
NT-proBNP (pg/mL, IQR)	1110.0 (330.1–3507.5)		2610.0 (555.5–9825.0)		0.024
LDL (mg/dL)	99.0	(80.0–121.0)	95.0	(72.0–118.0)	0.079
Albumin (g/dL)	4.0	(3.6–4.3)	3.8	(3.5–4.1)	0.024
Hba1c (%)	6.1	(5.6–6.9)	6.1	(5.7–7.0)	0.732
Creatinine (mg/dL)	1.0	(0.8–1.4)	1.2	(0.9–1.8)	< 0.001
eGFR (ml/min per 1.73m^2^)	67.7	(49.4–84.2)	59.5	(37.0–76.5)	0.001
Proteinuria (mg/g)	0.1	(0.1–0.3)	0.2	(0.1–0.5)	0.091
Medications					
Diuretics	413	(62.6)	135	(89.4)	< 0.001
MRA	96	(14.5)	77	(51.0)	< 0.001
β-blocker	352	(53.3)	123	(81.5)	< 0.001
ACEI or ARB	412	(62.4)	128	(84.8)	< 0.001
Anti-platelet agents	380	(57.6)	129	(85.4)	< 0.001
Anti-coagulants	80	(12.1)	43	(28.5)	< 0.001
Digoxin	58	(8.8)	32	(21.2)	< 0.001

Continuous data were expressed as median (IQR, interquartile range), and analyzed by the Mann-Whitney U test. Categorical data were expressed as number and percentage, and analyzed by the Chi-Square test

^a^Fried criteria (reference: [11])

^b^Rockwood frailty index (reference: [14].; [15])

*Abbreviations: HF* heart failure, *BMI* body mass index, *AF* atrial fibrillation, *COPD* chronic obstructive pulmonary disease, *CCI* Charlson Comorbidity Index, *IQR* interquartile range, *LVEF* left ventricular ejection fraction, *MNA-SF* mini-nutritional assessment-short form, *TUG* Timed Up and Go, HGS handgrip strength, *6 MW* 6-m walking, *NT-proBNP* N-terminal pro-B-type natriuretic peptide, *LDL* low density lipoprotein, *Hba1c* glycated hemoglobin, *eGFR* estimated glomerular filtration rate, *MRA* mineralocorticoid receptor antagonist, *ACEI* angiotensin-converting enzyme inhibitor, *ARB* angiotensin II receptor blockers, *SD* standard deviation. *eGFR* calculated using modified Modification diet of renal disease (MDRD) formula, was utilized to evaluate renal function

### Association between timed up and go test, handgrip strength, walking speed, and heart failure with reduced and preserved cardiac function

**Fig. 2:**
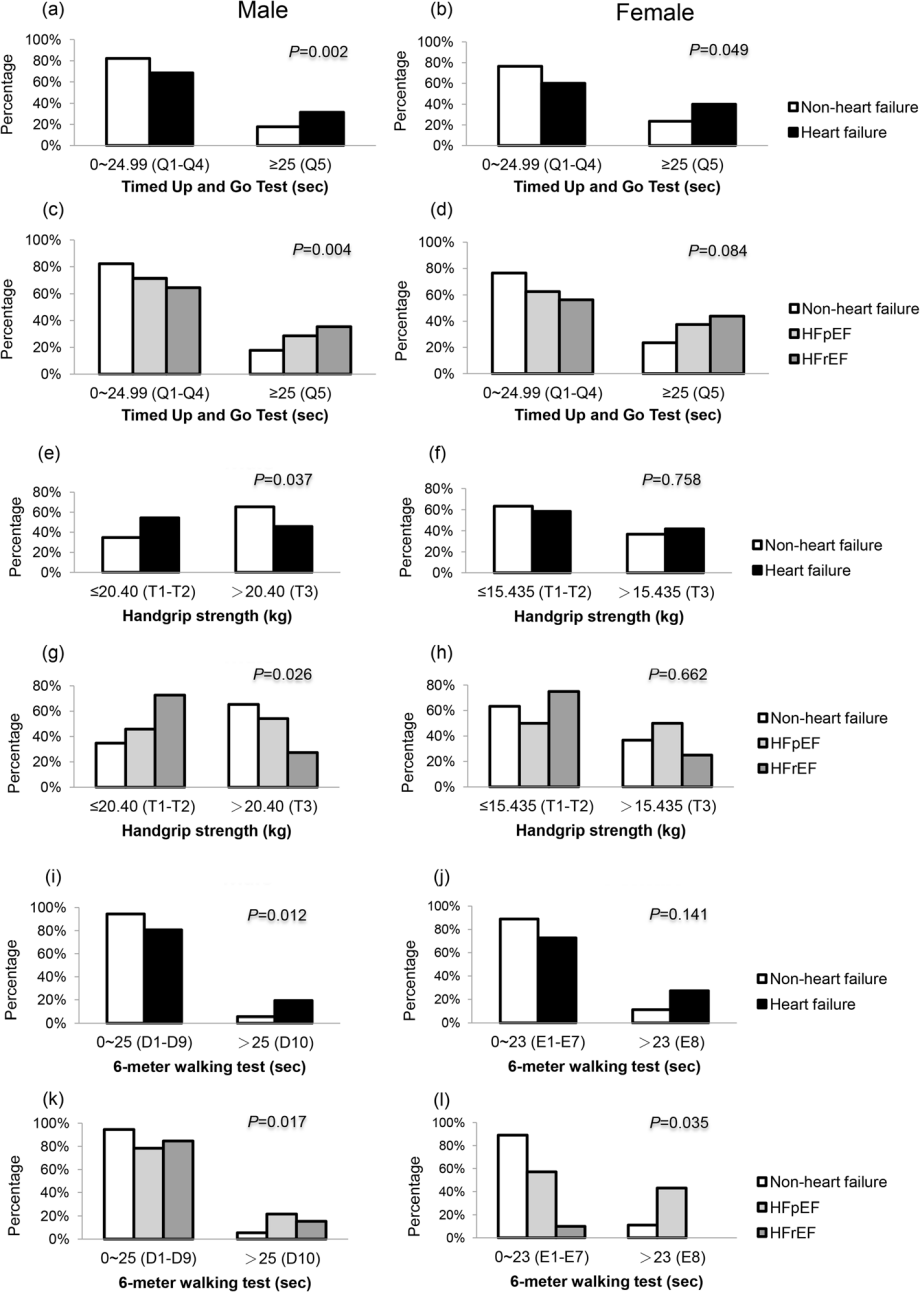
Physical functionality and heart failure (HF) with and without reduced ejection fraction (EF). (a,b) Representative image of Timed Up and Go (TUG) between HF and non-HF. (c,d) TUG among non-HF, HFpEF, and HFrEF. (e,f) Handgrip strength (HGS) between HF and non-HF. (g,h) HGS among non-HF, HFpEF, and HFrEF. (i,j) 6-m walking (6 MW) between HF and non-HF. (k,l) 6 MW among non-HF, HFpEF, and HFrEF. TUG values were divided into fifths both in men and women. HGS values were separated into tertiles both in men and women. 6 MW values were calculated into deciles with a cut-off point of 25 s in men and eight equal parts with a cut-off point of 23 s in women

In comparison with non-HF patients, HF patients had significantly longer TUG points (≥25 s, the last 20% of all patients) test (*p* = 0.002 in man, Fig. 2a; *p* = 0.049 in woman, Fig. 2b) with HFrEF patients having the highest TUG (≥25 s) points (Fig. 2c and d). For HGS, we found male HF patients had decreased handgrip (≤20.4 kgs, the first two-thirds of all male patients, *p* = 0.037) in comparison to non-HF patients, and HF severity was correlated with HGS (*p* = 0.026). However, there was no difference between HGS and the severity of HF in female patients (Fig. 2e-h). Likewise, it was found that male HF patients had a significantly higher percentage of longer 6 MW (> 25 s, the last 10% of male patients) test (*p* = 0.012, Fig. 2i), but no significant difference was found in females (Fig. 2j). When participants were divided into non-HF, HFpEF, and HFrEF groups, both HFpEF and HFrEF patients had significantly longer 6 MW tests in men (*p* = 0.017, Fig. 2k) and women (*p* = 0.035, Fig. 2l).

### NT-proBNP and timed up and go test and cardiac ejection fraction

We also examined the relationships among LVEF, NT-proBNP, and different functional status of TUG, HGS, and 6 MW in male HF and non-HF patients. When TUG was divided into quartiles in men, the LVEF was found to be associated with longer TUG in HF patients (supplemental data – Appendix 3a). Abnormal NT-proBNP was closely associated with severity of HF (median [IQR] = 831.4 [138.8 to 2803.3] pg/mL in non-HF, with 1110.0 [330.1 to 3507.5] pg/mL in HFpEF, and with 6190.0 [1490.0 to 15,616.0] pg/mL in HFrEF; *p* < 0.001), but was not associated with TUG, HGS and 6 MW. When HGS was divided into tertiles, HGS ≤10 ~ 20kgs in HF men was associated with the least LVEF (*p* < 0.001; supplemental data – Appendix 3e). For 6 MW, HF patients with short or long 6 MW of both genders had no significant difference in distinguishing between high and low LVEF (supplemental data – Appendix 3i-l).

### Joint effects of heart failure and poor handgrip strength associated with poor survival

**Fig. 3:**
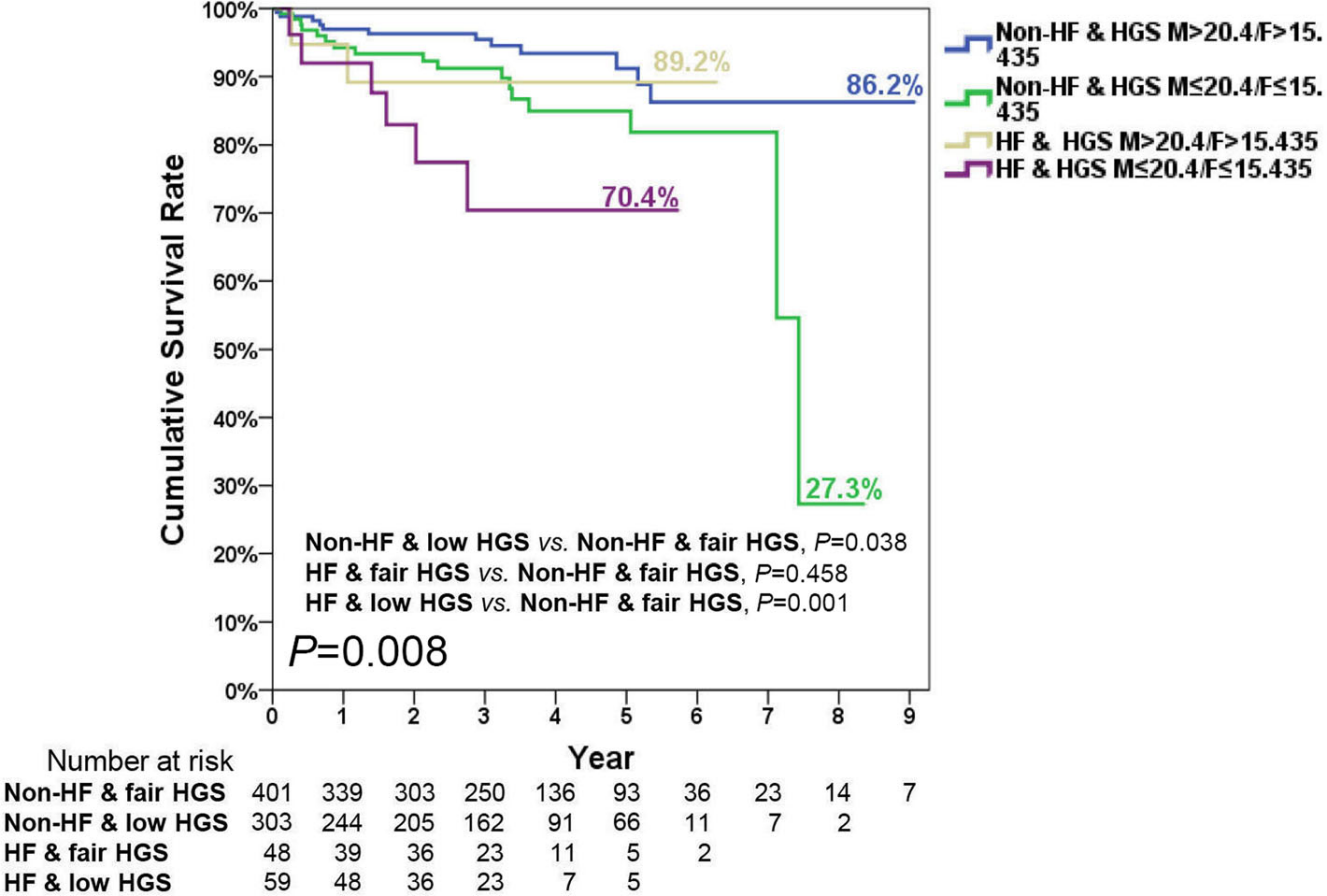
Kaplan-Meier survival curves for mortality stratified by the different levels of handgrip strength, heart failure (HF), and non-HF

When comparing baseline characteristics of survivors and non-survivors in patients with and without HF, it was found that non-survivors had a higher percentage of AF, COPD, CCI score, cardiac arrhythmia, poor HGS, and high Rockwood frailty index, as well as lower serum LDL, albumin, and eGFR, but higher serum creatinine. The non-survivor group had a significantly higher percentage of frailty which was defined by the Rockwood frailty index (91.3%) than that (78.1%) in the survivor group (*p* = 0.020). The percentage of drugs for HF was also high in the non-survivors (supplemental data – Appendix 4). During the follow-up period (median [quartiles] = 3.2 [1.5–4.8] years), the simple KM plots revealed that poor HGS (*p* = 0.010), longer TUG (*P* = 0.037), and one physical impairment (*p* = 0.029) were significantly correlated with poor cumulative survival (supplemental data – Appendix 5); however, the severity of HF showed no significant difference between survivors and non-survivors (supplemental – Appendix 6a). Among the participants with and without HF, it was shown that HF patients with decreased HGS had the poorest survival in the first 5 years, followed by non-HF patients with decreased HGS, HF with fair HGS, and non-HF patients with fair HGS, respectively (*p* = 0.008) (Fig. 3). There were no differences in the cumulative survival rates among the severity of HF, TUG, and at least one physical impairment (HTW) (supplemental data – Appendix 6b and 6c).

### High Rockwood frailty index, poor handgrip strength and one physical dysfunction associated with all-cause mortality

Using a univariate Cox regression model, HFrEF, CCI, TUG, HGS, one physical impairment, Rockwood frailty index, albumin, MRA, β-blocker, anti-coagulants, and digoxin were all significantly associated with mortality in all older patients (Table 2). In the multivariate Cox proportional hazards model, in all patients, a high Rockwood frailty index was independently associated with increased mortality (model 1, aHR = 1.05; 95% CI: 1.0004 to 1.10). In addition, the adjusted mortality HR was 3.42 with decreased HGS (95% CI: 1.03 to 11.40), 7.65 with use of mineralocorticoid receptor antagonist (95% CI: 2.22 to 26.32), and 1.26 with associated multi-comorbidities assessed by Charlson comorbidity index (95% CI: 1.05 to 1.51) (model 3, Table 2).

**Table 2:** Predictors of all-cause mortality in older adults

	Univariate analysis		Model 1		Model 2			Model 3			Model 4		
	HR	(95% CI)	HR	(95% CI)	HR	(95% CI)		HR	(95% CI)		HR	(95% CI)	
Age	1.01	(0.98–1.05)	0.99	(0.95–1.04)	1.00	(0.95–1.04)		1.02	(0.95–1.11)		0.99	(0.92–1.06)	
Male vs. Female	1.00	(0.62–1.62)	0.88	(0.46–1.68)	0.97	(0.50–1.89)		0.63	(0.16–2.41)		1.83	(0.53–6.35)	
Non-HF	ref.	–	ref	–	ref.	–		ref.	–				
HFpEF	0.80	(0.37–1.76)	0.19	(0.06–1.19)	0.28	(0.10–1.79)		0.30	(0.03–2.46)				
HFrEF	1.99	(1.05–3.78)*	0.19	(0.05–1.11)	0.27	(0.08–1.94)		0.97	(0.08–11.28)				
BMI	0.97	(0.92–1.02)											
CCI	1.26	(1.09–1.46)**	1.25	(1.03–1.50)*	1.06	(0.76–1.48)		1.26	(1.05–1.51)**				
Geriatric assessment													
MNA-SF	0.91	(0.83–1.01)											
Timed Up and Go test, sec													
< 25	ref.	–			ref.	–							
≥ 25	1.67	(1.02–2.71)*			1.02	(0.46–2.23)							
Handgrip strength, kg													
M > 20.4 / F > 15.435	ref.	–						ref.	–				
M ≤ 20.4 / F ≤ 15.435	2.34	(1.20–4.57)*						3.42	(1.03–11.40)*				
6-min walking test, sec													
M ≤ 25 / F ≤ 23	ref.	–											
M > 25 / F > 23	0.96	(0.22–4.11)											
HTW (0–3)													
0	ref.	–									ref.	–	
≥ 1	3.01	(1.07–8.50)*									3.22	(1.13–9.20)*	
Rockwood frailty index	1.06	(1.03–1.08)**	1.05	(1.0004–1.10)*									
Laboratory data													
Albumin, g/dL	0.30	(0.21–0.43)**	0.28	(0.16–0.49)**	0.31	(0.18–0.55)*		0.45	(0.17–1.20)				
Glucose, mg/dL	1.00	(1.00–1.00)	0.99	(0.99–0.998)*	0.99	(0.99–0.999)*		1.00	(0.99–1.01)				
eGFR, ml/min per 1.73m^2^	0.99	(0.98–0.998)*	1.00	(0.99–1.01)	0.99	(0.99–1.00)		1.00	(0.99–1.01)				
Medication													
MRA	4.61	(2.97–7.17)**	2.89	(1.45–5.73)**	3.30	(1.68–6.50)**		7.65	(2.22–26.32)**				
β-blocker	2.07	(1.22–3.50)**	1.33	(0.67–2.66)	1.42	(0.71–2.84)		0.67	(0.22–2.02)				
Anti-coagulants	1.84	(1.11–3.02)*	1.15	(0.46–2.84)	0.99	(0.40–2.45)		0.81	(0.10–6.37)				
Digoxin	4.75	(3.00–7.53)**	1.19	(0.85–4.29)	2.15	(0.95–4.83)		1.42	(0.32–6.27)				

*P < 0.05; **P < 0.001; Model 1, the Cox proportional hazards model was used to evaluate the association of all-cause mortality with Rockwood frailty index between the heart failure (HF) and non-HF older adults, and the multi-variate analysis was adjusted for age, gender, severity of HF, Charlson Comorbidity Index (CCI), laboratory tests, and medication. Model 2, the Cox proportional hazards model was used to evaluate the association of all-cause mortality with Timed Up and Go test between the HF and non-HF older adults, and the multi-variate analysis was adjusted for age, gender, severity of HF, CCI, laboratory tests, and medication. Model 3, the Cox proportional hazards model was used to evaluate the association of all-cause mortality with handgrip strength between the HF and non-HF older adults, and the multi-variate analysis was adjusted for age, gender, severity of HF, CCI, laboratory tests, and medication. Model 4, the Cox proportional hazards model was used to evaluate the association of all-cause mortality with at least one physical impairment (HTW means the summation of one abnormality of the HGS, TUG, and 6 MW) between the HF and non-HF older adults, and the multi-variate analysis was adjusted for age and gender only. *Abbreviations: BMI* body mass index; *MNA-SF* mini-nutritional assessment-short form; *MRA* mineralocorticoid receptor antagonist

## Discussion

In this retrospective cohort study, we examined the effects of physical functionality and frailty on older patients with and without HF. It was shown frailty and decreased physical functions were more prevalent in HF patients. The TUG test was found to be closely associated with the severity of HF in older adults, especially in men. Besides, frailty and HGS predicted prognosis in the patients, and there was a combined effect of HF and low HGS on survival. Recently, years lived with a disability is a crucial factor that must not be overlooked due to evidence of the importance of heterogeneity in individual frailty on the dynamics of mortality [17]. Therefore, the frailty index and sarcopenic phenotypes have been widely studied [18, 19]. The frailty has been hypothesized to implicate increased vulnerability to stressors (e.g. infection, injury, or even changes in medication) in older adults [11] due to dysregulation of interactions between multiple physiological regulatory functions, that in consequence compromises body homeostasis or resilience in the presence of stressors [20–22]. Clinically, to define frailty, it is based on phenotypical descriptions focusing on functional manifestations of frailty involving muscle weakness, reduced exercise tolerance, walking speed, physical activity, and weight loss [11], or deficit-based descriptions including disabilities, diseases, and laboratory examinations [23]. The possible causes of muscle strength decline include an imbalance between catabolic and anabolic signaling, chronic inflammation due to environmental and psychosocial factors, genetic factors, gut microbiomes, lifestyles, exercise, and nutrition [24–27]. In our studies, among different frailty assessment tools, the Fried criteria revealed a proportion of 52.5% of patients as frail, and the Rockwood frailty assessment tool found a proportion of 79.4% as frail (Table 1). These findings suggested that although the Fried criteria and Rockwood frailty assessment had different components, each was highly prevalent in elderly patients with HF, suggesting that these frailty assessment tools somehow reflect a common underlying phenotype. Our study also found that frailty was more common in patients with HF than in patients without HF. Besides, frailty assessed by the Rockwood index was associated with an increased hazard ratio of mortality. This finding was consistent with the results of recently published meta-analyses [28]. The findings of the present study have implications that in elderly patients with HF, it is important to accurately assess frailty status, because it can be targeted for treatment with various interventions, including rehabilitation, nutritional recommendations, reduction of polypharmacy, and HF self-care.

Several studies demonstrated that walking speed was a predictive outcome in patients with cardiovascular disease, including HF [8, 29]. Gait speed (GS) reflects the performance of the cardiorespiratory, nervous, and musculoskeletal systems, and is associated with mobility and exercise capacity in performing activities of daily living. These factors probably each play a role in the mechanism underlying the association between GS and HF severity. Further, Weiss et al. [30] applied instrumented TUG to older adults with lower functioning on transitioning from turning to sitting and found that TUG was a better physical performance predictor, and was associated with poorer motor and cognitive function, lower perceptual speed, and worse mobility disability. In fact, in a large cohort of Korean older people living in the community, it was found that slower speed of the TUG at baseline was associated with increased risk of myocardial infarction, CHF, and mortality with a clear dose-response relationship [31]. HGS, another indicator of frailty, correlates highly with the strength of elbow flexion, knee extension, and trunk extension [32] and it has been used to approximate overall muscle function, particularly in advanced HF patients with minimal tolerance of physical exertion and in hospitalized, deconditioned patients [33]. The potential link between lower HGS and reduced cardiac protection among older individuals is consistent with a pattern resembling concentric hypertrophy, which is characterized by higher LV mass, high LV mass to volume ratio (LVMVR), and an association with low HGS, according to results of the SmartHeart EPSRC program [34]. Moreover, in a Japanese study, HGS was found to be a significant prognostic index of survival in patients with HF [35]. Of note, in our study, it was found there were some gender differences between physical frailty parameters (e.g. HGS and 6 MW) and heart failure severity. Although factors associated with low HGS between older men and women are likely to different sociodemographic and behavioral factors, as well as health conditions [36], the exact reasons were not clear. It had been shown that among the older people in the community the risk factors for declined HGS seemed to be more lifestyle-related for women (e.g. smoking and stress), while for men more physically related factors (e.g. blood pressure, physical activity, and chronic disorders) were important [37]. Further research to evaluate relevant factors of grip and muscle strength (and biological vitality) for men and women with heart failure may be necessary.

In the survival analysis, TUG and 6 MW across subgroups of the severity of HF were not significantly predictive of mortality, whereas HF patients with poor HGS because of limitation of core activity, communication, mobility, and self-care were associated with all-cause death. This finding contradicted some previous investigations [34, 38–41]. In our study, reduced EF, poor handgrip strength with considerable use of mineralocorticoid receptor antagonist (MRA) and high CCI, and one abnormal physical function were all found to be associated with all-cause mortality. This raises the possibility that evaluation of individual physical function to predict outcome may accurately reflect the combined effect of multimorbidity, polypharmacy, and physical functionality acting synergistically on all-cause mortality.

### Clinical implication

The pathobiologies of frailty and HF share several common pathways, particularly a consistent correlation with inflammatory biomarkers as well as impaired mitophagy and mitochondrial dysfunction within cardiomyocytes and skeletal muscle, causing cell death and activation of innate immunity to induce chronic, low-grade systemic inflammation [42]. Our study findings indicated unmeasured heterogeneity of functional frailty in older HF population. As the physical functional decline in HF patients may be related to diverse socioeconomic and health profiles, improvements in prognosis may be achieved by adopting strategies aimed at preserving or enhancing physical functioning in order to prevent unplanned hospital admissions, reduce the cost of care, and decrease death rates.

### Strengths and limitations

The limitations of our study are as follows. First, this was a retrospective study. Therefore, longitudinal and prospective analyses are needed to further examine whether the physical decline associated with HF becomes worse and affects outcomes based on severity. Second, the average cut-off values of TUG, HGS, and 6 MW were arbitrary due to diverse physical functions in different groups of patients with major illnesses. Third, it would be useful, for further development, considering other outcomes such as 30 days hospital admission and quality of life for patients affected by HF. The strength of our study was that physical function (upper, low muscle strength, balance) was comprehensively investigated in older adults with HF or without HF.

## Conclusions

In conclusion, frailty and CHF were common in older patients, and the TUG test was closely associated with the severity of HF. Furthermore, physical functionality determined the prognosis with the worst survival in patients with concurrent physical limitations and HF. We suggest that heart function as well as frailty assessment should be evaluated in older patients to improve prognosis.

## Supplementary Information

**Additional file 1 **Supplementary information accompanies this paper at *BMC Geriatrics online.*

## Data Availability

The datasets used and analyzed during the current study are available from the corresponding author on reasonable request.

## Abbreviations

AF: Atrial fibrillation
BMI: Body mass index
CCI: Charlson Comorbidity Index
CHF: Chronic heart failure
CKD: Chronic kidney disease
COPD: Chronic obstructive pulmonary disease
eGFR: Estimated glomerular filtration rate
GDS-5: 5-item geriatric depression scale
GS: Gait speed
HbA1c: hemoglobin, hemoglobin A1c
HF: Heart failure
HGS: Handgrip strength
HFrEF: Heart failure with reduced ejection fraction
HFpEF: Heart failure with preserved ejection fraction
ICD-9-CM: International Classification of Diseases, Ninth Revision, Clinical Modification
IRB: Institutional Review Board
IQR: Interquartile range
LDL: Low-density lipoprotein
LVEF: Left ventricular ejection fraction
MMSE: Mini-mental state examination
MRA: Mineralocorticoid receptor antagonist
MNA-SF: Mini-Nutritional Assessment-Short Form
NT-proBNP: N-terminal pro-B-type natriuretic peptide
TUG: Timed Up and Go
6 MW: 6-m walking
SD: Standard deviation
