# Supplementary material for: Physical frailty and long-term mortality in older people with chronic heart failure with preserved and reduced ejection fraction: a retrospective longitudinal study
Source: BMC Geriatr. 2021 Feb 1;21:92. doi: 10.1186/s12877-020-01971-4 (PMC7849094; doi:10.1186/s12877-020-01971-4)
Supplement: Supplementary file 1 — Additional file 1 Supplementary information accompanies this paper at BMC Geriatrics online. [file 12877_2020_1971_MOESM1_ESM.pdf]

Supplemental data

Physical frailty and long-term mortality in older people with chronic heart failure with preserved and reduced ejection fraction: a retrospective longitudinal study

Shuo-Chun Weng[1,2,3], Chu-Sheng Lin[2,4], Der-Cherng Tarng[1,5,6,7,8] and Shih-Yi Lin[1,2,9,*]

[1]Institute of Clinical Medicine, School of Medicine, National Yang-Ming University, Taipei, Taiwan. [2]Center for Geriatrics and Gerontology, Taichung Veterans General Hospital, Taichung, Taiwan. [3]Division of Nephrology, Department of Internal Medicine, Taichung Veterans General Hospital, Taichung, Taiwan. [4]Department of Family Medicine, Taichung Veterans General Hospital, Taichung, Taiwan.

[5]Department and Institute of Physiology, National Yang-Ming University, Taipei, Taiwan. [6]Division of Nephrology, Department of Medicine, Taipei Veterans General Hospital, Taipei, Taiwan. [7]Center for intelligent Drug Systems and Smart Bio-devices (IDS2B), Taiwan. [8]Department of Biological Science and Technology, College of Biological Science and Technology, National Chiao Tung University, Hsinchu, Taiwan. [9]Division of Endocrinology and Metabolism, Department of Internal Medicine, Taichung Veterans General Hospital, Taichung, Taiwan.

Supplemental data – **Appendix 1.** ATC code for drugs for heart failure

Supplemental data – **Appendix 2.** Prevalence of heart failure (HF), HF with preserved ejection fraction (HFpEF), and HF with reduced ejection fraction (HFrEF)

Supplemental data – **Appendix 3.** Correlations among (a-d) Timed Up and Go Test (TUG), (e-h) handgrip strength (HGS), (i-l) 6-meter walking test (6MW), left ventricular ejection fraction (LVEF) and N-terminal pro-B-type natriuretic peptide

(NT-proBNP) in both genders.

Supplemental data – **Appendix 4.** Comprehensive geriatric assessment and all-cause mortality

Supplemental data – **Appendix 5.** Kaplan-Meier survival curves for different levels of (a) handgrip strength (HGS); (b) Timed Up and Go test (TUG); (c) physical functionality stratified by the summation (HTW) of abnormal HGS, TUG, and 6-meter walking test (6MW).

Supplemental data – **Appendix 6.** Kaplan-Meier survival curves for (a) non-HF, HFpEF, and HFrEF. (b) mortality stratified by the different levels of TUG, HF and, non-HF. (c) mortality stratified by the different levels of summation of abnormal HGS, TUG, and 6MW (HTW), HF and, non-HF.

Supplemental data – **Appendix 1.** ATC code for drugs for heart failure

	ATC codes	Health insurance codes
Diuretics		
Hychlozide TAB 25mg	C03AA03	NC00141100
Dithiazide TAB 25mg	C03AA03	AC030981G0
Trichlormethiazide TAB 2mg	C03AA06	AC46028100
Metolazone TAB 0.5mg	C03BA08	AC50772100
Furosemide TAB 40mg	C03CA01	AC226411G0
Furosemide inj 20mg	C03CA01	AB27928212
Furosemide inj 20mg	C03CA01	BC22840212
Furosemide oral solution 10mg/ml	C03CA01	AB44046157
Amiloride 5mg/Hydrochlorothiazide 50mg TAB (Amizide)	C03EA01	AC29320100
Mineralocorticoid receptor antagonist (MRA)		
Spironolactone TAB 25mg	C03DA01	AC185691G0
Eplerenone TAB 50mg (Inspra)	C03DA04	BC24306100
Beta-blocker		
Propranolol HCl TAB 40mg	C07AA05	AB198751G0
Propranolol TAB 10mg	C07AA05	AB230371G0
Atenolol TAB 100mg	C07AB03	AB343591G0
Atenolol TAB 50mg	C07AB03	BC221601G0
Acebutolol HCL TAB 400mg	C07AB04	BC06049100
Bisoprolol TAB 1.25mg	C07AB07	BC24039100
Bisoprolol TAB 5mg	C07AB07	BC17125100
Labetalol HCL TAB 200mg (Trandate)	C07AG01	BC24532100
Labetalol HCL inj 25mg (Trandate)	C07AG01	AC32350221
Carvedilol TAB 6.25mg (Dilatrend)	C07AG02	BC22071100
Carvedilol TAB 25mg (Dilatrend)	C07AG02	BC20452100
Angiotensin-converting enzyme inhibitors and angiotensin II receptor blockers		
Captopril TAB 25mg	C09AA01	BC19632100
Captopril TAB 25mg	C09AA01	AC348801G0
Enalapril Maleate TAB 20mg (Renitec)	C09AA02	AC394141G0
Lisinopril TAB 10mg	C09AA03	AC42824100
Lisinopril TAB 10mg	C09AA03	BC22152100
Perindopril TAB 5mg (Acertil)	C09AA04	BC24725100
Ramipril Cap 2.5mg (Ramitace)	C09AA05	AC46995100
Accupril TAB 10mg	C09AA06	BC22124100
Imidapril HCL TAB 10mg	C09AA16	AC43951100
Amlodipine besylate 5mg + Benazepril HCL 10mg TAB	C09BB	AB46742100
Losartan potassium TAB 50 mg	C09CA01	AC45203100

Losartan TAB 50mg (Cozaar)	C09CA01	BC21914100
Valsartan TAB 80mg	C09CA03	BC23373100
Telmisartan TAB 80mg	C09CA07	BC23161100
Olmesartan Medoxomil TAB 20mg	C09CA08	BC24497100
Azilsartan medoxomil TAB 40mg (EDARBI)	C09CA09	BC25756100
Losartan potassium 50mg/ Hydrochlorothiazide 12.5mg TAB (Hysartan F.C.)	C09DA01	AA48757100
Losartan100mg/ Hydrochlorothiazide12.5mg TAB (Hyzaar)	C09DA01	BC24643100
Valsartan 80mg/ Amlodipine besylate 6.94mg TAB	C09DB01	AC57797100
Amlodipine besylate 10mg/ Valsartan 160mg/ Hydrochlorothiazide 25mg TAB (Exforge HCT)	C09DX01	BC25421100
Amlodipine 5mg/ Telmidartan 80mg TAB (Twynsta)	C09DB04	BC25446100
Amlodipine 5mg/ Olmesartan medoxomil 20mg TAB (Sevikar)	C09DB02	BC24929100
Amlodipine 5mg/ Hydrochlorothiazide 12.5mg/ Olmesartan medoxomil 20mg TAB (Sevikar HCT)	C09DX03	BC25492100
Angiotensin receptor-neprilysin inhibitor		
Sacubitril 97mg/ Valsartan 103mg TAB (Entresto 200mg)	C09DX04	BC26671100
Sacubitril 49mg/ Valsartan 51mg TAB (Entresto 100mg)	C09DX04	BC26672100
Nitrate		
Nitroglycerin TAB 0.6mg (nitrostat)	C01DA02	BC20802100
Isosorbide dinitate TAB 10mg	C01DA08	AC27438100
Isosorbide dinitate inj 10mg (Angidil)	C01DA08	AC44974229
Isosorbide dinitrate inj 10mg (Isoket)	C01DA08	BC24062229
Isosorbide mononitrate TAB 20mg (Isormol)	C01DA14	AC311681G0
Isosorbide-5-mononitrate TAB 20mg (Ismo-20)	C01DA14	BC112121G0
Isosorbide-5-mononitrate TAB 60mg (Ismo-60 CR)	C01DA14	BC20554100
Inotropic agents		

Dopamine HCL inj 200mg/5mL/Amp	C01CA04	AC32704221
Dopamine HCL/Glucose inj 3mg/mL (Gipamine)	C01CA04	AC46457263
Anti-platelet agents		
Clopidofrel hydrogen sulfate TAB 75mg (Thrombifree FC)	B01AC04	AA48730100
Clopidogrel TAB 75mg (Plavix FC)	B01AC04	BC22932100
Ticlopidine HCL TAB 100mg (Licodin FC)	B01AC05	AB31596100
Aspirin TAB 100mg (Aspirin FC)	N02BA01	A024465100
Aspirin Cap 100mg (Bokey EM)	B01AC06	AC373441G0
Aspirin Cap 100mg (Ascotyl EM)	B01AC06	AC436631G0
Acetylsalicyclic acid 25mg/ Dipyridamole 200mg CAP (Aggrenox Medified Release)	B01AC07, B01AC06, B01AC30	BC23919100
Dipyriamole 75mg (Perzin FC)	B01AC07	AC019411G0
Dipyriamole 25mg	B01AC07	AC136341G0
Traditional anti-coagulants		
Warfarin sodium 1mg (Cofarin)	B01AA03	AC43862100
Warfarin 5mg (Orfarin)	B01AA03	BC23572100
New anti-platelet agents		
Ticagrelor TAB 90mg (Brilinta)	B01AC24	BC25691100
Dabigatran CAP (Pradaxa)	B01AE07	BC25458100
Dabigatran etexilate mesilate CAP 110mg (Pradaxa)	B01AE07	BC25459100
Rivaroxaban TAB 10mg (Xarelto FC)	B01AF01	BC25129100
Rivaroxaben TAB 15mg (Xarelto FC)	B01AF01	BC25648100
Apixaban TAB 5mg (Eliquis FC)	B01AF02	BC26133100
Edoxaban TAB 60mg (Lixiana FC)	B01AF03	BC26599100
Fondaparinus sodium inj 2.5mg/0.5mL (Arixtra)	B01AX05	BC25126206
Digoxin		
Digoxin TAB 0.25mg (Lanoxin)	C01AA05	BC09554100
Digoxin inj 0.5mg/2mL/amp (Lanoxin)	C01AA05	BC09714212
Digoxin inj 50ug/mL (Cardiacin Elixir)	C01AA05	AC47126151

Supplemental data – **Appendix 2.** Prevalence of heart failure (HF), HF with preserved ejection fraction (HFpEF), and HF with reduced ejection fraction (HFrEF)

Year	Heart failure
2009	32.3%
2010	29.5%
2011	35.2%
2012	29.1%
2013	25.5%
2014	27.3%
2015	31.0%
2016	31.3%
2017	39.5%
2018	35.3%
Total	28.5%

	HF with preserved EF	HF with reduced LVEF
2009	12.9%	9.7%
2010	15.8%	5.3%
2011	14.3%	9.9%
2012	8.9%	9.9%
2013	9.5%	9.1%
2014	11.2%	8.8%
2015	11.7%	10.6%
2016	3.0%	13.4%
2017	5.3%	21.1%
2018	11.8%	17.6%
Total	10.4%	9.7%

		Group 1<br>n (%)	Group 2<br>n (%)	Group 3<br>n (%)	Group 4<br>n (%)	Total<br>n (%)
2009	Non-HF	3 (60.0)	12 (92.3)	5 (62.5)	4 (80.0)	24 (77.4)
	HFpEF	1 (20.0)	1 (7.7)	2 (25.0)	0 (0.0)	4 (12.9)
	HFrEF	1 (20.0)	0 (0.0)	1 (12.5)	1 (20.0)	3 (9.7)
		5	13	8	5	31
2010	Non-HF	40 (76.9)	21 (87.5)	10 (83.3)	4 (57.1)	75 (78.9)
	HFpEF	9 (17.3)	2 (8.3)	2 (16.7)	2 (28.6)	15 (15.8)
	HFrEF	3 (5.8)	1 (4.2)	0 (0.0)	1 (14.3)	5 (5.3)
		52	24	12	7	95
2011	Non-HF	25 (71.4)	15 (71.4)	16 (80.0)	13 (86.7)	69 (75.8)
	HFpEF	6 (17.1)	4 (19.0)	2 (10.0)	1 (6.7)	13 (14.3)

	HFrEF	4 (11.4)	2 (9.5)	2 (10.0)	1 (6.7)	9 (9.9)
		35	21	20	15	91
2012	Non-HF	19 (86.4)	101 (89.4)	114 (75.5)	11 (68.8)	245 (81.1)
	HFpEF	1 (4.5)	6 (5.3)	18 (11.9)	2 (12.5)	27 (8.9)
	HFrEF	2 (9.1)	6 (5.3)	19 (12.6)	3 (18.8)	30 (9.9)
		22	113	151	16	302
2013	Non-HF	19 (73.1)	122 (86.5)	151 (70.6)	190 (90.0)	482 (81.4)
	HFpEF	1 (3.8)	9 (6.4)	39 (18.2)	7 (3.3)	56 (9.5)
	HFrEF	6 (23.1)	10 (7.1)	24 (11.2)	14 (6.6)	54 (9.1)
		26	141	214	211	592
2014	Non-HF	9 (60.0)	69 (82.1)	134 (74.0)	190 (85.6)	402 (80.1)
	HFpEF	2 (13.3)	10 (11.9)	28 (15.5)	16 (7.2)	56 (11.2)
	HFrEF	4 (26.7)	5 (6.0)	19 (10.5)	16 (7.2)	44 (8.8)
		15	84	181	222	502
2015	Non-HF	7 (63.6)	70 (82.4)	118 (78.1)	18 (66.7)	213 (77.7)
	HFpEF	1 (9.1)	8 (9.4)	21 (13.9)	2 (7.4)	32 (11.7)
	HFrEF	3 (27.3)	7 (8.2)	12 (7.9)	7 (25.9)	29 (10.6)
		11	85	151	27	274
2016	Non-HF	0 (0.0)	20 (80.0)	17 (94.4)	19 (86.4)	56 (83.6)
	HFpEF	1 (50.0)	1 (4.0)	0 (0.0)	0 (0.0)	2 (3.0)
	HFrEF	1 (50.0)	4 (16.0)	1 (5.6)	3 (13.6)	9 (13.4)
		2	25	18	22	67
2017	Non-HF	0 (0.0)	9 (90.0)	11 (68.8)	8 (66.7)	28 (73.7)
	HFpEF	0 (0.0)	1 (10.0)	1 (6.3)	0 (0.0)	2 (5.3)
	HFrEF	0 (0.0)	0 (0.0)	4 (25.0)	4 (33.3)	8 (21.1)
		0	10	16	12	38
2018	Non-HF	1 (50.0)	7 (100.0)	2 (66.7)	2 (40.0)	12 (70.6)
	HFpEF	0 (0.0)	0 (0.0)	0 (0.0)	2 (40.0)	2 (11.8)
	HFrEF	1 (50.0)	0 (0.0)	1 (33.3)	1 (20.0)	3 (17.6)
		2	7	3	5	17
Total	Non-HF	123 (72.4)	446 (85.3)	578 (74.7)	459 (84.7)	1606 (79.9)
	HFpEF	22 (12.9)	42 (8.0)	113 (14.6)	32 (5.9)	209 (10.4)
	HFrEF	25 (14.7)	35 (6.7)	83 (10.7)	51 (9.4)	194 (9.7)
		170	523	774	542	2009

Group 1. Older adults attending community-based healthcare screening programs

Group 2. Outpatient clinic of the geriatric department

Group 3. Inpatient of the geriatric department

Group 4. Residents in a veterans' home

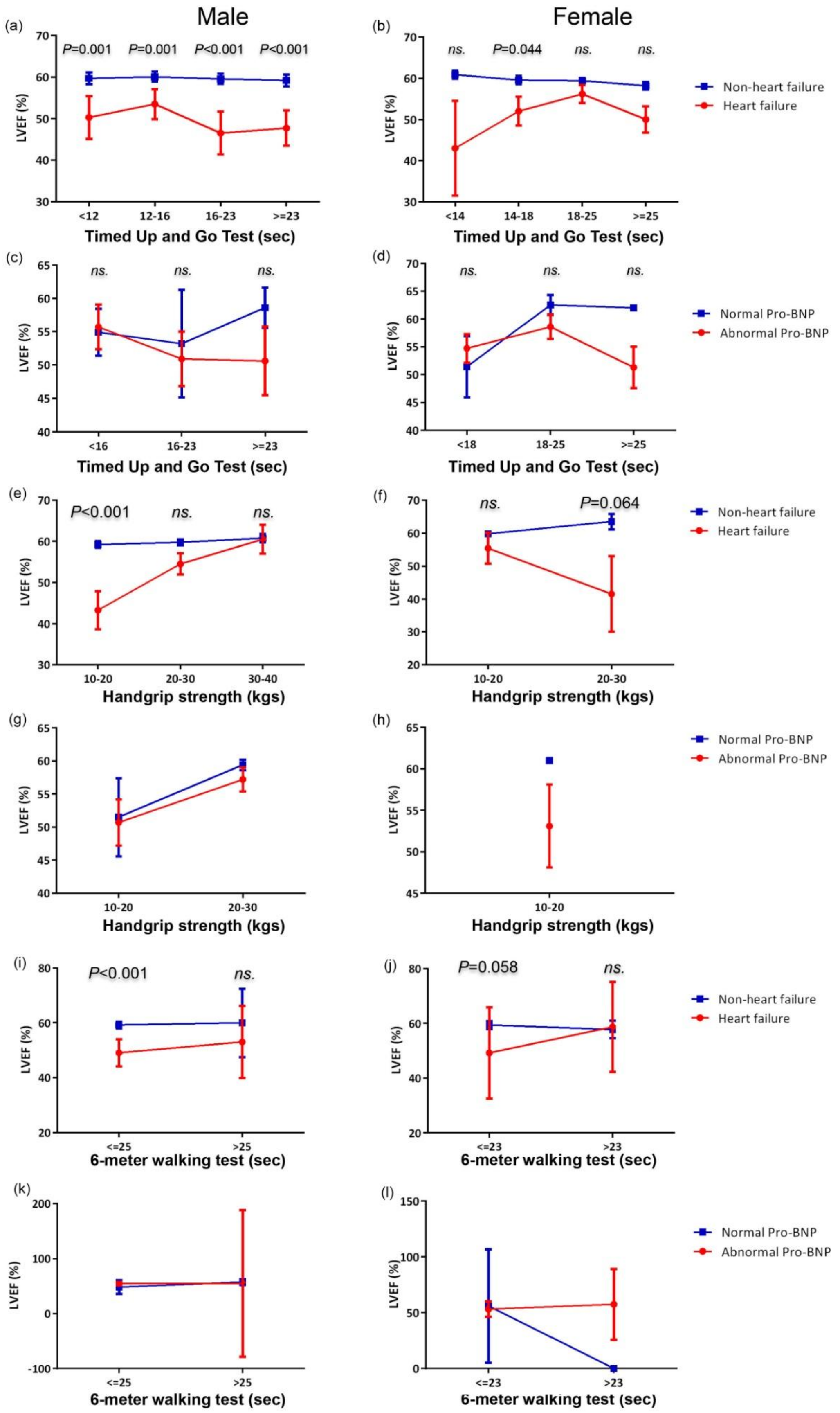

Supplemental data – **Appendix 3.** Correlations among (a-d) Timed Up and Go Test (TUG), (e-h) handgrip strength (HGS), (i-l) 6-meter walking test (6MW), left ventricular ejection fraction (LVEF) and N-terminal pro-B-type natriuretic peptide (NT-proBNP) in both genders.

Supplemental data – **Appendix 4.** Comprehensive geriatric assessment and all-cause mortality

	Alive (*n* = 731)	Death (*n* = 80)	*P* value
Mean age (years)	81.5(74.8-85.4)	82.5 (74.8-86.7)	0.263
Male	494(67.6)	56(70.0)	0.753
BMI (kg/m^2)	24.0(21.8-26.8)	24.0(21.2-26.8)	0.809
Comorbidities			
Diabetes mellitus	387(52.9)	38(47.5)	0.420
Hypertension	622(85.1)	70(87.5)	0.680
Hyperlipidemia	315(43.1)	36(45.0)	0.835
Cardiovascular disease	195(26.7)	20(25.0)	0.850
AF	21(2.9)	7(8.8)	0.015
Myocardial infarction	78(10.7)	8(10.0)	1.000
COPD	287(39.3)	44(55.0)	0.009
CCI	1.0(1.0-2.0)	2.0(1.0-3.0)	0.030
Heart condition			0.114
Non-HF	598(81.8)	62(77.5)	
HFpEF	80(10.9)	7(8.8)	
HFrEF	53(7.3)	11(13.8)	
LVEF	59.0(54.0-61.0)	58.5(54.0-60.3)	0.467
Cardiac arrhythmia	46(6.3)	12(15.0)	0.008
Geriatric assessment			
MNA-SF (0 to 14) (SD)	13.0(11.0-14.0)	13.0(11.0-14.0)	0.388
Timed Up and Go test			0.189
<25 sec	573(78.4)	57(71.3)	
≥25 sec	158(21.6)	23(28.8)	
Timed Up and Go test (sec)			0.167
M<12 / F<14	148(20.2)	12(15.0)	
M12-15.9 / F14-17.9	194(26.5)	15(18.8)	
M16-22.9 / F18-24.9	204(27.9)	29(36.3)	
M≥23 / F≥25	185(25.3)	24(30.0)	
Handgrip strength (kg)			0.037
M≤20.4 / F≤15.435	311(42.6)	50(62.2)	
M>20.4 / F>15.435	420(57.4)	30(37.8)	
6-meter walking test (sec)			1.000
M≤25 / F≤23	659(90.2)	73(91.7)	
M>25 / F>23	72(9.8)	7(8.3)	
Fried frailty index[a]			0.423
Robust	89(12.2)	6(7.3)	

Pre-frail	255(34.9)	35(43.9)	
Frail	386(52.9)	39(48.8)	
Rockwood frailty index[b]	27.8(22.6-34.2)	32.9(26.3-39.5)	<0.001
Non-frail	11(1.5)	0(0.0)	0.020
Pre-frail	149(20.4)	7(8.8)	
Frail	571(78.1)	73(91.3)	
Abnormal THW			
0	376(51.4)	22(27.8)	0.096
≥1	355(48.6)	58(72.2)	
Laboratory data			
NT-proBNP (pg/mL, IQR)	1240.0 (330.0-4775.0)	2100.0 (625.4-7676.5)	0.105
LDL (mg/dL)	99.0(80.0-120.0)	87.0(66.0-119.0)	0.009
Albumin (g/dL)	4.0(3.6-4.3)	3.6(3.2-3.9)	<0.001
Hba1c (%)	6.1(5.6-6.9)	6.2(5.6-7.0)	0.995
Creatinine (mg/dL)	1.1(0.8-1.4)	1.3(0.9-2.1)	<0.001
eGFR (ml/min per 1.73m^2)	67.0(49.1-83.5)	54.5(28.3-76.7)	<0.001
Proteinuria (mg/g, IQR)	0.1(0.1–0.3)	0.2(0.1–0.4)	0.951
Medications			
Diuretics	470(64.3)	78(97.5)	<0.001
MRA	131(17.9)	42(52.5)	<0.001
β-blocker	413(56.5)	62(77.5)	<0.001
ACEI or ARB	481(65.8)	59(73.8)	0.191
Anti-platelet agents	459(62.8)	50(62.5)	1.000
Anti-coagulants	102(14.0)	21(26.3)	0.006
Digoxin	62(8.5)	28(35.0)	<0.001

Continuous data were expressed as median (IQR, interquartile range), and analyzed by the Mann-Whitney U test. Categorical data were expressed as number and percentage, and analyzed by the Chi-Square test.

[a]Fried criteria (reference: Fried LP, Tangen CM, Walston J, Newman AB, Hirsch C, Gottdiener J, et al. Frailty in older adults: evidence for a phenotype. J Gerontol A Biol Sci Med Sci. 2001;56(3):M146–56.)

[b]Rockwood frailty index (reference: Peña FG, Theou O, Wallace L, Brothers TD, Gill TM, Gahbauer EA, et al. Comparison of alternate scoring of variables on the performance of the frailty index. BMC Geriatr. 2014;14:25.; Orkaby AR, Lunetta KL, Sun FJ, Driver JA, Benjamin EJ, Hamburg NM, et al. Cross-sectional association of frailty and arterial stiffness in community-dwelling older adults: the Framingham Heart Study. J Gerontol A Biol Sci Med Sci. 2019;74(3):373-379.)

Abbreviations: HF heart failure, *BMI* body mass index, *AF* atrial fibrillation, *COPD* chronic obstructive pulmonary disease, *CCI* Charlson Comorbidity Index, *IQR* interquartile range, *LVEF* left ventricular ejection fraction, *MNA-SF* mini-nutritional assessment-short form, *TUG* Timed Up and Go, HGS handgrip strength, *6MW* 6-meter walking, *NT-proBNP* N-terminal pro-B-type natriuretic peptide, *LDL* low density lipoprotein, *Hba1c* glycated hemoglobin, *eGFR* estimated glomerular filtration rate, *MRA* mineralocorticoid receptor antagonist, *ACEI* angiotensin-converting enzyme inhibitor, *ARB* angiotensin II receptor blockers, *SD* standard deviation. *eGFR* calculated

using modified Modification diet of renal disease (MDRD) formula, was utilized to evaluate renal function.

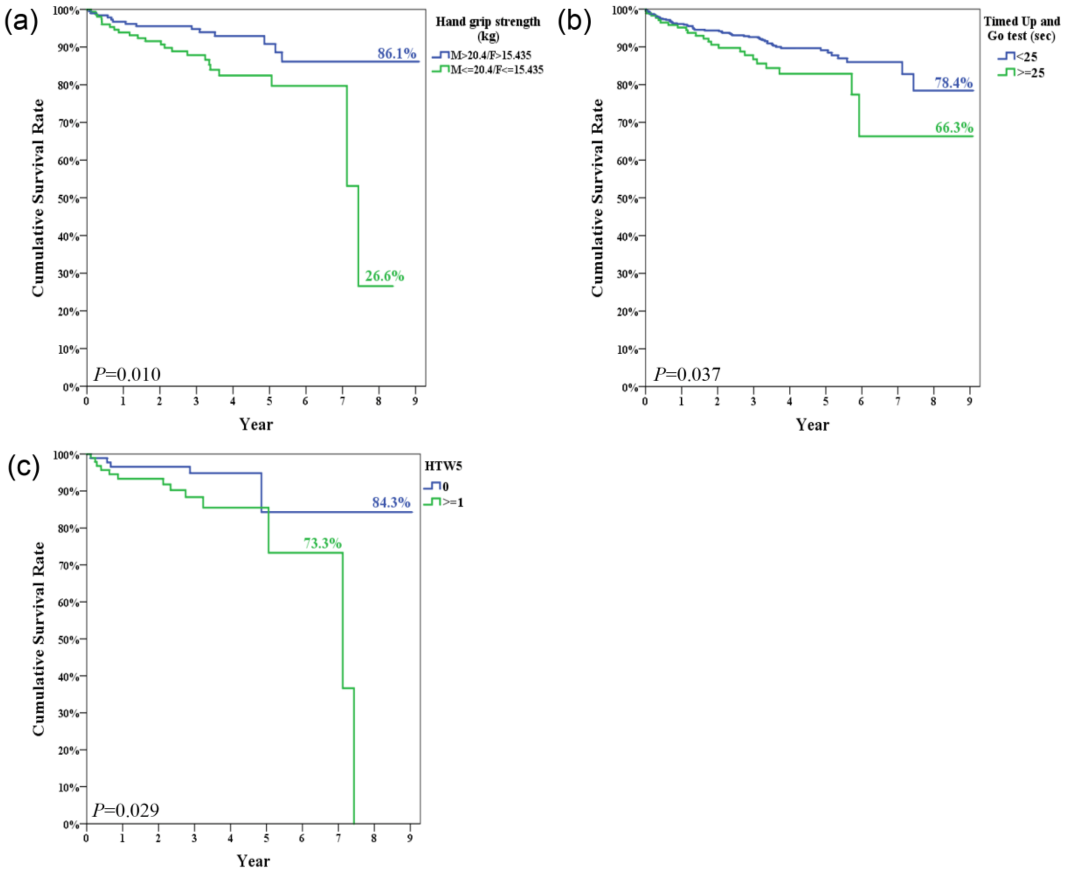

Supplemental data – **Appendix 5.** Kaplan-Meier survival curves for different levels of (a) handgrip strength (HGS); (b) Timed Up and Go test (TUG); (c) physical functionality stratified by the summation (HTW) of abnormal HGS, TUG, and 6-meter walking test (6MW).

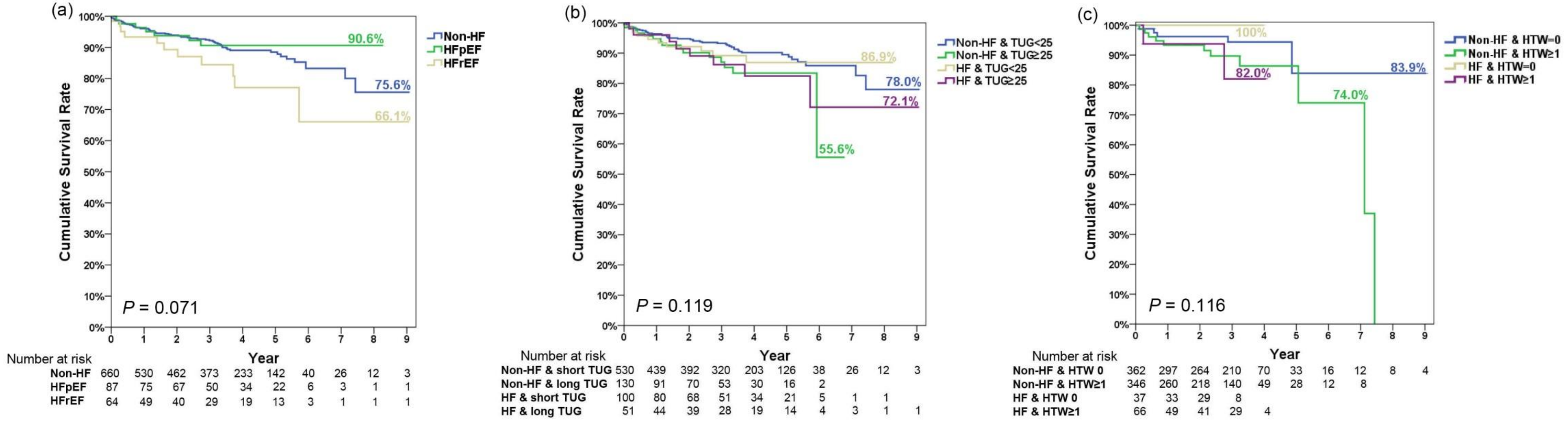

Supplemental data – **Appendix 6.** Kaplan-Meier survival curves for (a) non-HF, HFpEF, and HFrEF. (b) mortality stratified by the different levels of TUG, HF and, non-HF. (c) mortality stratified by the different levels of summation of abnormal HGS, TUG, and 6MW (HTW), HF and, non-HF.
